# Association between video laryngoscope blade geometry and first-pass success during prehospital intubation: a prospective bicentric observational study

**DOI:** 10.1186/s12873-026-01701-w

**Published:** 2026-07-23

**Authors:** Christoph Jänig, Benedikt Harmuth, Holger Meyer, Andreas Schwartz, Maja Iversen, Marc Royko, Andreas Garcia Bardon, Daniel Schroeder, Tobias Grübl, Martin Schiffarth, Willi Schmidbauer, Tim Piepho

**Affiliations:** 1 Department of Anaesthesia and Critical Care Medicine, Bundeswehr Central Hospital Koblenz, Rübenacher Straße 170, 56072 Koblenz, Germany; 2 Department of Anaesthesia and Critical Care Medicine, Bundeswehr Hospital Hamburg, Hamburg, Germany; 3 ADAC Air Rescue gGmbH, Weßling, Germany; 4 Department of Anaesthesia and Critical Care Medicine, Hospital of the Brothers of Mercy Trier, Trier, Germany

**Keywords:** Video laryngoscopy, Helicopter emergency medical services, Prehospital airway management, Endotracheal intubation, First-pass success, Blade geometry

## Abstract

**Background:**

Endotracheal intubation (ETI) is a cornerstone of advanced prehospital airway management but remains associated with considerable procedural risk, particularly when multiple intubation attempts are required. First-pass success (FPS) is an established quality indicator because repeated attempts increase the risk of hypoxaemia, aspiration, oesophageal intubation, haemodynamic instability and cardiac arrest [1–4]. Although video laryngoscopy (VL) is recommended by international airway guidelines [5–8], evidence regarding the optimal blade geometry for prehospital airway management remains limited. We investigated the association between video laryngoscope blade geometry and FPS in a physician-staffed helicopter emergency medical service (HEMS).

**Methods:**

We conducted a prospective bicentric observational study at two German physician-staffed HEMS bases between January 2017 and December 2019. Consecutive prehospital ETIs performed using the C-MAC^®^ PM video laryngoscope were prospectively documented. Blade selection was at the discretion of the treating physician. The primary outcome was FPS. Secondary outcomes included glottic visualisation, intubation duration, number of attempts, airway-related adverse events and video laryngoscopy-associated technical problems.

**Results:**

A total of 283 video laryngoscopic intubations were analysed. Overall first-pass success (FPS) was 71.0% (201/283). Blade-specific FPS rates were 72.0% for Macintosh size 3, 72.6% for Macintosh size 4 and 61.9% for the hyper angulated D-Blade, without significant differences between blade geometries (*p* = 0.601). Adequate glottic visualisation (Cormack–Lehane grade I–II) was achieved in 96.8% of patients. Mean intubation duration was 42.5 ± 34.2 s. Airway-related adverse events occurred in 5.7% of patients and video laryngoscopy-associated technical problems in 12.6%. In an exploratory multivariable analysis, blade geometry was not independently associated with FPS, whereas difficult laryngoscopy conditions, poor glottic visualisation and technical problems were associated with failed first-pass intubation.

**Conclusions:**

Video laryngoscopy provided excellent glottic visualisation irrespective of blade geometry. Neither univariable nor exploratory multivariable analyses demonstrated an independent association between blade geometry and first-pass success. Instead, difficult airway conditions, poor glottic visualisation and video laryngoscopy-associated technical problems appeared to exert a greater influence on procedural success. Given the observational design and limited number of hyper angulated blade intubations, these findings should be regarded as hypothesis-generating and require confirmation in larger prospective multicentre studies incorporating standardised difficult-airway assessment.

## Introduction

Advanced airway management remains one of the most critical interventions in emergency medicine. Endotracheal intubation (ETI) provides definitive airway protection and enables adequate oxygenation and ventilation in patients with major trauma, respiratory failure, cardiac arrest or severely impaired consciousness [9]. In contrast to elective airway management performed in the operating room, prehospital ETI is frequently undertaken under adverse environmental conditions, including restricted patient access, suboptimal positioning, limited personnel, ongoing resuscitation and airway contamination. These factors contribute to substantially higher complication rates and make successful airway management considerably more challenging [2, 10].

Repeated intubation attempts have consistently been associated with increased rates of hypoxaemia, aspiration, oesophageal intubation, hypotension and cardiac arrest [1–4]. Consequently, first-pass success (FPS) has evolved into one of the most important quality indicators in emergency airway management and is increasingly used as a benchmark in both clinical studies and quality assurance programmes [1, 2]. Improving FPS has therefore become a principal objective of contemporary airway management strategies.

During the past decade, video laryngoscopy (VL) has fundamentally changed airway management in both hospital and prehospital settings. Multiple randomised controlled trials and meta-analyses have demonstrated that VL improves glottic visualisation and reduces oesophageal intubation compared with direct laryngoscopy, particularly in anticipated difficult airways [5, 11–14]. These findings have led several national and international societies—including the American Society of Anaesthesiologists (ASA), the Difficult Airway Society (DAS) and other expert groups—to recommend video laryngoscopy as the preferred primary device whenever available, especially in patients at increased risk of difficult intubation [6–8]. Nevertheless, current guidelines provide little guidance regarding the optimal blade geometry for emergency airway management.

Modern video laryngoscopes are available with different blade geometries, most commonly conventional Macintosh-style blades and hyper angulated blades. Macintosh-style blades resemble conventional direct laryngoscope blades and permit immediate conversion to direct laryngoscopy when optical visualisation becomes impaired. Hyper angulated blades, such as the C-MAC^®^ D-Blade, were specifically developed to improve glottic visualisation in anatomically difficult airways but require dedicated tube shaping and modified insertion techniques [15, 16]. Although several comparative studies have demonstrated superior glottic visualisation with hyper angulated blades, improvements in procedural success have been inconsistent, particularly when experienced airway providers perform the intubation [13, 17–20].

Most published studies have compared video laryngoscopy with direct laryngoscopy or evaluated different video laryngoscope systems in operating rooms, emergency departments or intensive care units. In contrast, evidence specifically addressing blade selection within the same video laryngoscope platform under real-world prehospital conditions remains scarce.

International emergency medical systems differ considerably with respect to provider qualification and organisational structure. In many Anglo-American emergency medical systems, advanced airway management is primarily performed by paramedics working in ground-based emergency medical services. In contrast, German-speaking countries operate physician-based emergency medical systems in which both helicopter emergency medical services (HEMS) and ground-based emergency medical services are routinely staffed by emergency physicians, most commonly board-certified anaesthesiologists with extensive experience in advanced airway management [21–23]. These structural differences may influence both first-pass success and the relative importance of blade geometry.

The participating HEMS services represent highly specialised physician-led systems in which advanced airway management is performed by experienced anaesthesiologists using a single video laryngoscope platform. This setting provides a unique opportunity to evaluate whether blade geometry independently influences first-pass success when provider expertise is relatively homogeneous.

Therefore, the primary objective of this prospective bicentric observational study was to investigate the association between video laryngoscope blade geometry and first-pass success during prehospital endotracheal intubation in a physician-staffed HEMS system. Secondary objectives included comparisons of glottic visualisation, intubation duration, number of attempts, blade changes, airway-related adverse events and video laryngoscopy-associated technical problems. We hypothesised that, within an experienced physician-staffed HEMS system, blade geometry would have only a limited influence on first-pass success compared with patient- and procedure-related factors.

## Materials and methods

### Study design and reporting

This prospective, bicentric observational cohort study was conducted to investigate the association between video laryngoscope blade geometry and first-pass success during prehospital endotracheal intubation (ETI) in a physician-staffed helicopter emergency medical service (HEMS). The study adhered to the Strengthening the Reporting of Observational Studies in Epidemiology (STROBE) statement for observational research.

### Study setting

The study was performed between January 2017 and December 2019 at two physician-staffed German HEMS bases:

- Christoph 23, Koblenz
- Christoph 29, Hamburg

Both services operate within the German physician-based emergency medical system, in which emergency physicians are routinely dispatched by helicopter and, depending on operational requirements, also participate in ground-based emergency responses. In contrast to Anglo-American EMS systems, advanced airway management is predominantly performed by board-certified physicians, most commonly anaesthesiologists with extensive experience in emergency airway management [21–24].

Christoph 23 is based at the Bundeswehr Central Hospital Koblenz and provides emergency medical coverage for both urban and rural regions within an operational radius of approximately 70 km, performing approximately 2,000 missions annually.

Christoph 29 is based at the Bundeswehr Hospital Hamburg and operates within the German civil protection system. During the study period, both HEMS bases used identical airway equipment and comparable standard operating procedures for prehospital airway management.

### Participants

All consecutive patients undergoing prehospital ETI using the C-MAC^®^ PM video laryngoscope (Karl Storz SE & Co. KG, Tuttlingen, Germany) during the study period were eligible for inclusion.

#### Inclusion criteria

- Prehospital endotracheal intubation
- Use of the C-MAC^®^ PM video laryngoscope
- Prospective documentation using the standardized airway documentation form

#### Exclusion criteria

- Intubation performed using direct laryngoscopy
- Missing documentation precluding assessment of the primary endpoint

During the study period, 285 prehospital intubations were documented. Two patients were excluded because direct laryngoscopy was used, leaving 283 video laryngoscopic intubations available for analysis.

Both adult and paediatric patients were eligible. No age restrictions were applied.

### Airway device and blade selection

The C-MAC^®^ PM video laryngoscope served as the standard video laryngoscope at both participating HEMS bases throughout the study period.

The following reusable blade geometries were available:

- Macintosh size 3
- Macintosh size 4
- D-Blade (hyper angulated)
- Miller size 1
- Miller size 2

Blade selection was entirely left to the discretion of the treating emergency physician. No predefined blade selection algorithm or institutional recommendation existed. Instead, physicians selected blade geometry according to their clinical judgement, taking into account patient characteristics, anticipated airway difficulty and procedural circumstances.

Because blade allocation was not randomised, the present study should be interpreted as an observational comparison reflecting routine clinical practice rather than an interventional evaluation of blade performance.

### Data collection

Data were prospectively collected immediately after each mission using a standardized airway documentation form routinely employed at both participating HEMS bases.

The documentation form included:

- Patient demographics
- Indication for intubation
- Blade selected for the first attempt
- Cormack–Lehane grade
- Number of intubation attempts
- Blade changes
- Difficult airway characteristics
- Use of adjunctive airway manoeuvres (e.g. stylet, BURP manoeuvre)
- Airway-related adverse events
- Video laryngoscopy-associated technical problems.

Whenever activated, the C-MAC^®^ PM system automatically generated digital video recordings of the intubation procedure. Video files were stored locally and subsequently matched with the corresponding documentation forms.

Of the 283 included intubations:

- 161 (56.9%) video recordings were retrievable;
- 148 (52.3%) complete recordings covering the entire intubation procedure were available for assessment of intubation duration.

Incomplete recordings were excluded from time analysis.

### Definitions

#### Intubation attempt

An intubation attempt was defined as insertion of the video laryngoscope blade beyond the incisors.

Removal of either the laryngoscope blade or the endotracheal tube from the oral cavity before successful tracheal tube placement was regarded as a failed attempt.

#### First-pass success

The primary endpoint, first-pass success (FPS), was defined as successful tracheal intubation after a single insertion of the video laryngoscope without removal of either the blade or endotracheal tube.

This strict definition is consistent with contemporary airway research and quality improvement initiatives emphasising the importance of uninterrupted first-attempt success [1–4].

#### Intubation duration

Intubation duration was measured from passage of the video laryngoscope blade beyond the incisors until removal of the blade following confirmed tracheal tube placement.

Only complete video recordings were used for this analysis.

#### Glottic visualisation

Glottic visualisation was graded according to the Cormack–Lehane classification.

For statistical analyses, grades I and II were considered adequate visualisation, whereas grades III and IV were classified as difficult glottic visualisation.

#### Difficult airway conditions

Potentially difficult airway conditions were documented according to the treating physician’s clinical assessment.

These included:

- Cervical spine immobilisation
- Limited cervical mobility
- Airway contamination (blood, vomitus or secretions)
- Restricted patient access
- Environmental limitations.

No standardized difficult airway assessment tool, such as the LEMON or MACOCHA score, was prospectively applied [25, 26].

#### Airway-related adverse events

Patient-related adverse events comprised:

- Oxygen desaturation
- Aspiration
- Oesophageal intubation
- Mucosal injury
- Dental injury.

Video laryngoscopy-associated technical problems included:

- Camera fogging
- Contamination of the optical system
- Monitor fogging
- Glare due to sunlight
- Technical device malfunction.

### Outcomes

#### Primary outcome

The primary outcome was first-pass success according to video laryngoscope blade geometry.

#### Secondary outcomes

Secondary outcomes included:

- Cormack–Lehane grade
- Intubation duration
- Number of intubation attempts
- Blade changes
- Airway-related adverse events
- Video laryngoscopy-associated technical problems.

### Statistical analysis

Statistical analyses were performed using IBM SPSS Statistics Version 26.0 (IBM Corp., Armonk, NY, USA).

Categorical variables are presented as absolute frequencies and percentages.

Continuous variables are reported as mean ± standard deviation (SD) or median with range, as appropriate.

Comparisons between blade geometries were performed using:

- Pearson’s χ² test for categorical variables;
- Kruskal–Wallis test for continuous variables not meeting assumptions of normality.

Because only three Miller blade intubations were documented, these cases were excluded from comparative statistical analyses involving blade geometry.

No formal sample size calculation was performed because all consecutive patients meeting the inclusion criteria during the predefined study period were included.

A two-sided p-value < 0.05 was considered statistically significant.

An exploratory multivariable logistic regression analysis was performed to assess independent associations with first-pass success. Because of the limited number of D-Blade intubations, the regression model was restricted to a small number of clinically relevant covariates and should be regarded as hypothesis-generating rather than confirmatory.

### Ethics

The study was conducted in accordance with the principles of the Declaration of Helsinki.

According to the responsible institutional ethics committee, the project constituted prospective quality assurance and therefore did not require formal ethical approval under applicable national regulations.

All patient data were anonymised before analysis.

Because of the observational study design and the emergency setting, the requirement for individual informed consent was waived.

## Results

### Study population

Between January 2017 and December 2019, a total of 285 prehospital endotracheal intubations were prospectively documented at the two participating physician-staffed HEMS bases. Two patients were excluded because direct laryngoscopy rather than video laryngoscopy was used, leaving 283 video laryngoscopic intubations for the final analysis.

Of the included procedures, 239 (84.5%) were performed at Christoph 23 (Koblenz) and 44 (15.5%) at Christoph 29 (Hamburg). Video recordings were available for 161 procedures (56.9%), of which 148 (52.3%) were complete and suitable for analysis of intubation duration.

### Indications for endotracheal intubation

Prehospital endotracheal intubation was performed for a broad spectrum of emergency conditions, including traumatic injuries, out-of-hospital cardiac arrest, respiratory failure, impaired consciousness and other medical emergencies requiring definitive airway protection. As the primary objective of this study was to evaluate the association between blade geometry and procedural success, indication-specific subgroup analyses were not performed.

### Blade selection

Five different blade geometries were used during the study period. Macintosh size 3 represented the most frequently selected blade (186/283; 65.7%), followed by Macintosh size 4 (73/283; 25.8%). The hyper angulated D-Blade was used in 21 patients (7.4%), whereas Miller blades were selected in only three cases (1.1%). Blade distribution was comparable between the two participating HEMS bases, with Macintosh blades representing the preferred first-line device at both centres (Table 1).

**Table 1:** Distribution of video laryngoscope blades

Blade geometry	*n*	%
Macintosh size 3	186	65.7
Macintosh size 4	73	25.8
D-Blade	21	7.4
Miller size 1	2	0.7
Miller size 2	1	0.4
Total	283	100

### First-pass success

Overall, successful tracheal intubation during the first attempt was achieved in 201 of 283 patients, corresponding to an overall first-pass success (FPS) of 71.0%.

**Fig. 1:**
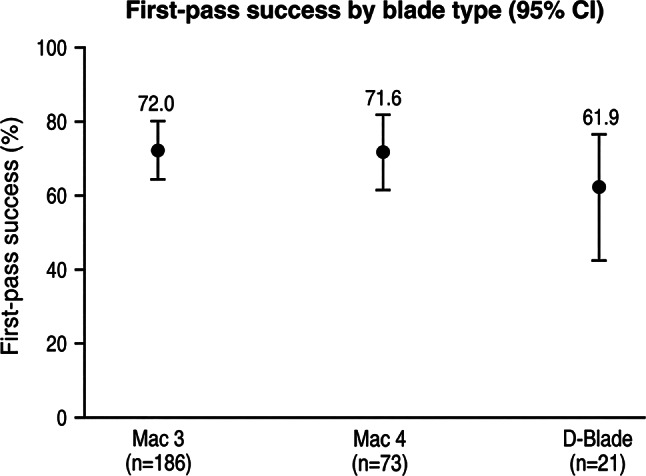
First-pass success by blade type. Proportion of successful first-pass intubations according to blade geometry. Error bars represent 95% confidence intervals

Blade-specific FPS rates were 72.0% for Macintosh size 3, 72.6% for Macintosh size 4 and 61.9% for the hyper angulated D-Blade. Because only three Miller blade intubations were performed, these cases were excluded from comparative statistical analyses. No statistically significant difference in FPS was observed between Macintosh size 3, Macintosh size 4 and D-Blade intubations (χ² test, *p* = 0.601; Table 2 + Fig. 1).

**Table 2:** First-pass success according to blade geometry

Blade geometry	Total (*n*)	FPS (*n*)	FPS (%)
Macintosh size 3	186	134	72.0
Macintosh size 4	73	53	72.6
D-Blade	21	13	61.9
Miller blades	3	1	33.3

### Glottic visualisation

Overall glottic visualisation was excellent. Adequate glottic visualisation (Cormack–Lehane grade I–II) was achieved in 274 of 283 patients (96.8%), whereas only nine patients (3.2%) demonstrated grade III or IV visualisation. No statistically significant difference in adequate glottic visualisation was observed between blade geometries (*p* = 0.360).

### Intubation duration

**Fig. 2:**
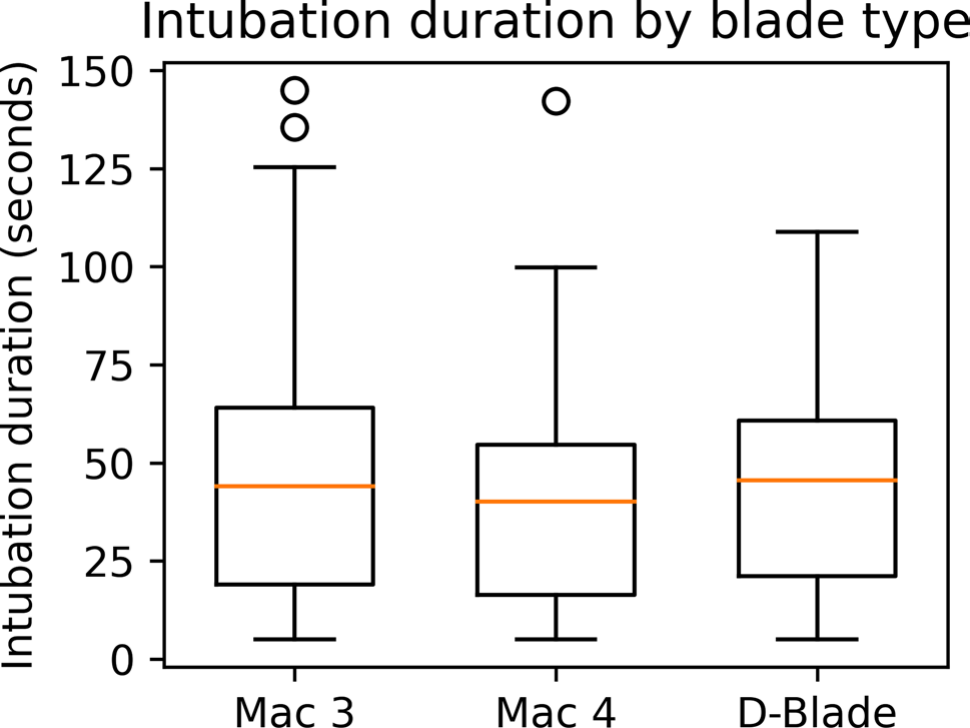
Intubation times. Intubation time by blade type (p = 0.307)

Analysis of intubation duration was possible in the 148 patients with complete video recordings. The overall mean intubation time was 42.5 ± 34.2 s, with a median duration of 30.5 s (range 8–235 s; Fig. 2).

Mean intubation duration according to blade geometry is presented in Table 3. Although D-Blade intubations tended to require longer intubation times, no statistically significant difference was observed between blade geometries (Kruskal–Wallis test, *p* = 0.177).

**Table 3:** Intubation duration according to blade geometry

Blade geometry	Mean ± SD (s)	Median (Range), s
Macintosh size 3	44.1 ± 37.1	32 (8–235)
Macintosh size 4	35.9 ± 27.6	26 (9–146)
D-Blade	54.1 ± 33.6	47 (15–138)

### Number of intubation attempts

Successful tracheal intubation was achieved on the first attempt in 201 patients (71.0%), on the second attempt in 69 patients (24.4%), and on the third attempt in nine patients (3.2%). In four patients (1.4%), video laryngoscopic tracheal intubation was unsuccessful despite repeated attempts.

Alternative airway management was required in these four patients, including placement of a supraglottic airway device in two cases and establishment of a surgical airway in two cases. No conversion to direct laryngoscopy was documented (Table 4).

**Table 4:** Number of intubation attempts

Outcome	*n*	%
Successful on first attempt	201	71.0
Successful on second attempt	69	24.4
Successful on third attempt	9	3.2
Intubation unsuccessful	4	1.4

### Airway-related adverse events

Patient-related adverse events occurred in 16 patients (5.7%), comprising 18 individual events. Oxygen desaturation was the most frequently documented complication, followed by aspiration and oesophageal intubation. One mucosal injury was recorded, whereas no dental injuries occurred (Table 5).

An additional 18 procedures (6.4%) were complicated by unexpected procedural problems unrelated to blade geometry, including difficulties during tube preparation, stylet handling, cuff defects and subglottic stenosis.

**Table 5:** Airway-related adverse events

Adverse event	*n* (%)
Oxygen desaturation	7 (2.5)
Aspiration	5 (1.8)
Oesophageal intubation	5 (1.8)
Mucosal injury	1 (0.4)
Dental injury	0

Airway-related adverse events were more frequent after failed first-pass intubation. Among patients with successful first-pass intubation, patient-related adverse events occurred in 3 of 201 cases (1.5%), whereas 13 of 82 patients (15.9%) without first-pass success experienced at least one airway-related adverse event (Fisher’s exact test, *p* < 0.001).

Videolaryngoscopy-associated technical problems were also more common when first-pass intubation failed. Technical problems occurred in 7 of 201 patients (3.5%) with first-pass success compared with 25 of 82 patients (30.5%) without first-pass success.

### Videolaryngoscopy-associated technical problems

Videolaryngoscopy-associated technical problems were documented in 32 procedures (12.6%), comprising 36 individual events. The most frequent problems were fogging of the optical system (17 cases; 6.0%), contamination of the camera by blood or secretions (9 cases; 3.2%) and impaired monitor visibility due to sunlight (6 cases; 2.1%). Monitor fogging and device malfunction each occurred in two procedures (0.7%).

Among patients with documented videolaryngoscopy-associated technical problems, 25 of 32 (78.1%) required at least one additional intubation attempt.

### Exploratory multivariable analysis

To address potential confounding, an exploratory multivariable logistic regression analysis was performed after exclusion of the three Miller blade cases. First-pass success served as the dependent variable. Independent variables included blade geometry, Cormack–Lehane grade, difficult laryngoscopy conditions and videolaryngoscopy-associated technical problems.

After adjustment, use of the D-Blade was not independently associated with first-pass success (OR 0.92, 95% CI 0.29–2.86; *p* = 0.884). In contrast, Cormack–Lehane grade III–IV (OR 0.13, 95% CI 0.02–0.74; *p* = 0.021), difficult laryngoscopy conditions (OR 0.32, 95% CI 0.17–0.61; *p* < 0.001) and videolaryngoscopy-associated technical problems (OR 0.09, 95% CI 0.03–0.23; *p* < 0.001) were independently associated with reduced odds of first-pass success (Table 6).

**Table 6:** Exploratory multivariable logistic regression analysis of factors associated with first-pass success

Variable	Odds ratio	95% CI	*p*-value
D-Blade vs. Macintosh	0.92	0.29–2.86	0.884
Cormack–Lehane III–IV	0.13	0.02–0.74	0.021
Difficult laryngoscopy conditions	0.32	0.17–0.61	< 0.001
Technical problems during VL	0.09	0.03–0.23	< 0.001

## Discussion

This prospective bicentric observational study investigated the association between videolaryngoscope blade geometry and first-pass success (FPS) during prehospital endotracheal intubation in a physician-staffed helicopter emergency medical service (HEMS). Four principal findings emerged. First, videolaryngoscopy provided excellent glottic visualisation irrespective of blade geometry, with adequate Cormack–Lehane grades in almost 97% of patients. Second, no statistically significant association between blade geometry and FPS was observed in either the univariable or exploratory multivariable analyses. Third, difficult laryngoscopy conditions, poor glottic visualisation and videolaryngoscopy-associated technical problems were independently associated with failed first-pass intubation. Finally, patient- and procedure-related factors appeared to have a greater influence on procedural success than blade geometry itself.

### First-pass success in the context of previous literature

First-pass success has become one of the most important quality indicators in emergency airway management because repeated attempts are associated with increased rates of hypoxaemia, aspiration, oesophageal intubation and cardiovascular complications [1–4, 27]. Accordingly, contemporary airway guidelines emphasise maximising first-pass success as a central objective of emergency airway management [6–8].

The overall FPS of 71.0% observed in the present study is lower than that reported by several physician-staffed prehospital airway registries although differences in first-pass definitions considerably limit direct comparison. Hossfeld et al. described substantially higher first-pass success using the same C-MAC PM videolaryngoscope in German physician-led emergency medical services [28], while Scandinavian and Swiss HEMS systems have reported FPS rates exceeding 85–90% [23, 24, 29]. Several factors may explain these differences.

First, our study applied a particularly stringent definition of FPS. Any removal of either the laryngoscope or the tracheal tube before successful tracheal placement was considered a failed first attempt. Such definitions differ across the literature and may substantially influence reported FPS rates [1, 27].

Second, our cohort reflects routine clinical practice in a real-world HEMS environment. Restricted patient access, ongoing cardiopulmonary resuscitation, contamination of the airway by blood or gastric contents, adverse weather conditions and limited positioning opportunities are well recognised challenges of prehospital airway management and have previously been associated with lower intubation success rates [31–33].

Third, the patient population likely differed from previously published cohorts. Although indication-specific subgroup analyses were beyond the scope of the present study, prehospital intubation in our cohort was frequently performed in critically ill or severely injured patients. Previous studies have demonstrated that first-pass success differs considerably between trauma, medical emergencies and out-of-hospital cardiac arrest [28, 34].

### Blade geometry does not appear to be an independent determinant of first-pass success

The primary objective of the present study was to determine whether videolaryngoscope blade geometry independently influences first-pass success.

Neither the univariable comparison nor the exploratory multivariable logistic regression demonstrated an independent association between D-Blade use and FPS. After adjustment for difficult laryngoscopy conditions, Cormack–Lehane grade and videolaryngoscopy-associated technical problems, blade geometry remained unrelated to first-pass success.

These findings should be interpreted cautiously. The number of D-Blade intubations was relatively small, and blade selection was entirely at the discretion of the treating physician. Hyperangulated blades are preferentially selected when a difficult airway is anticipated. Consequently, confounding by indication cannot be excluded and probably explains at least part of the lower unadjusted first-pass success observed with the D-Blade.

Nevertheless, the multivariable analysis strengthens the principal conclusion of the study by suggesting that patient- and procedure-related factors rather than blade geometry itself are the dominant determinants of successful prehospital intubation.

### Glottic visualisation versus successful tube delivery

One of the most notable findings is the discrepancy between excellent glottic visualisation and comparatively modest first-pass success. Although adequate Cormack–Lehane grades were obtained in almost all patients, nearly one-third required more than one intubation attempt.

This observation supports previous studies demonstrating that video laryngoscopy improves visualisation of the laryngeal inlet without necessarily increasing first-pass success [5, 13, 14]. Kelly and Cook have previously highlighted that video laryngoscopy frequently allows clinicians to “see more than they can intubate” [11]. Successful tracheal intubation depends not only on visualisation of the glottis but also on tube delivery, stylet configuration, hand–eye coordination and sufficient working space.

The present findings further support this concept. Poor glottic visualisation (Cormack–Lehane grade III–IV) remained independently associated with failed first-pass intubation, but excellent visualisation alone was clearly insufficient to guarantee procedural success.

### Technical problems are major determinants of procedural success

A particularly important finding of this study is the strong association between video laryngoscopy-associated technical problems and first-pass success. Technical problems were documented in 12.6% of procedures and were independently associated with failed first-pass intubation in the multivariable analysis.

Most technical problems resulted from camera contamination by blood or secretions, fogging of the optical system or impaired screen visibility due to sunlight. These findings are consistent with previous reports identifying optical impairment as one of the principal limitations of video laryngoscopy outside the operating room [31].

Unlike elective airway management, prehospital environments frequently offer limited opportunities for patient positioning or adequate suction. Consequently, optimisation of secretion management, anti-fogging strategies and operator training in troubleshooting optical problems may contribute more to improving first-pass success than preferential use of a particular blade geometry.

### Clinical implications

The present findings have several practical implications.

First, blade selection should primarily be guided by anticipated airway anatomy and operator familiarity rather than by the expectation that a particular blade geometry inherently improves first-pass success.

Second, structured airway training should emphasise tube delivery techniques, management of contaminated airways and troubleshooting of video laryngoscopy-associated technical problems.

Finally, quality improvement initiatives should focus on modifiable procedural factors. Our exploratory multivariable analysis suggests that optimisation of difficult airway management and reduction of technical problems may provide greater improvements in first-pass success than changes in blade selection alone.

### Future research

Future prospective multicentre studies should incorporate standardised difficult-airway assessment using validated instruments such as the LEMON or MACOCHA score [25, 26], balanced recruitment across participating centres and sufficiently large sample sizes to permit adequately powered multivariable analyses. In addition, indication-specific subgroup analyses separating trauma, medical emergencies and out-of-hospital cardiac arrest may help identify patient populations that derive the greatest benefit from specific video laryngoscope blade geometries.

### Limitations

Several limitations should be considered when interpreting the findings of this study.

First, the observational study design precludes causal inference. Blade selection was entirely at the discretion of the treating physician without predefined selection criteria or randomisation. Consequently, hyper angulated blades were likely preferentially selected in patients with anticipated difficult airways, introducing the potential for confounding by indication. Therefore, the observed differences in first-pass success may primarily reflect differences in airway complexity rather than the intrinsic performance of individual blade geometries.

Second, although an exploratory multivariable logistic regression analysis was performed to account for potential confounding, the results should be interpreted with caution. The relatively small number of D-Blade intubations limited statistical power and restricted the complexity of the regression model. Consequently, residual confounding cannot be excluded, and the multivariable analysis should be regarded as hypothesis-generating rather than confirmatory.

Third, no validated difficult-airway assessment tool, such as the LEMON or MACOCHA score, was prospectively recorded. Difficult airway conditions were documented according to the treating physician’s clinical judgement, preventing objective quantification of baseline airway complexity and limiting adjustment for patient-related risk factors.

Fourth, several potentially relevant variables—including the specific indication for intubation, provider-specific airway experience, annual intubation volume and detailed physiological characteristics of the patients—were either unavailable or incompletely documented and therefore could not be incorporated into the multivariable analysis. These unmeasured variables may have influenced procedural success and contributed to residual confounding.

Although the documentation form included the clinical indication for intubation, the available electronic dataset did not contain a sufficiently robust categorical indication variable suitable for reliable subgroup analysis. Therefore, indication-specific comparisons between trauma, medical emergencies and out-of-hospital cardiac arrest were not performed. Future studies should prospectively capture predefined indication categories to permit stratified analyses.

Fifth, recruitment was markedly unbalanced between the participating HEMS bases, with approximately 85% of patients originating from a single centre. Although both services followed comparable organisational structures, standard operating procedures and used identical airway equipment, this imbalance may limit the external validity of the findings.

Sixth, only 148 of the 283 video laryngoscopic procedures were available as complete video recordings for measurement of intubation duration. Although no systematic differences between patients with and without complete recordings were identified, incomplete video availability may have introduced selection bias into time-related analyses.

Finally, the study was conducted exclusively within German physician-staffed helicopter emergency medical services, where advanced airway management is routinely performed by experienced anaesthesiologists. Consequently, the present findings may not be directly transferable to ground-based emergency medical services or healthcare systems in which advanced airway management is primarily performed by paramedics or providers with different levels of airway experience.

Despite these limitations, the study has several important strengths. It represents one of the few prospective multicentre investigations comparing video laryngoscope blade geometries within a single device platform under real-world prehospital conditions. Standardised prospective data collection, inclusion of consecutive patients and the additional exploratory multivariable analysis strengthen the validity of the findings and provide a comprehensive assessment of factors associated with first-pass success during physician-staffed prehospital airway management.

## Conclusions

In this prospective bicentric observational study conducted within a physician-staffed helicopter emergency medical service, video laryngoscopy provided consistently excellent glottic visualisation across all blade geometries. Neither univariable comparisons nor exploratory multivariable analyses demonstrated an independent association between video laryngoscope blade geometry and first-pass success.

Instead, difficult laryngoscopy conditions, poor glottic visualisation and video laryngoscopy-associated technical problems were independently associated with failed first-pass intubation, suggesting that patient- and procedure-related factors exert a greater influence on procedural success than blade geometry alone. These findings emphasise that optimisation of prehospital airway management should focus not only on device selection but also on structured difficult-airway assessment, technical proficiency, management of contaminated airways and systematic training in video laryngoscopy under realistic prehospital conditions.

Given the observational design, physician-directed blade selection and the limited number of hyper angulated blade intubations, causal inferences cannot be drawn. The exploratory multivariable analysis should therefore be considered hypothesis-generating. Larger prospective multicentre studies incorporating standardised difficult-airway assessment, balanced patient recruitment and adequately powered multivariable analyses are required to determine whether blade geometry independently influences first-pass success in defined prehospital patient populations.

## Data Availability

The datasets generated and analyzed during the current study are available from the corresponding author on reasonable request.
